# Molecular Mechanisms Involved in Neural Substructure Development during Phosphodiesterase Inhibitor Treatment of Mesenchymal Stem Cells

**DOI:** 10.3390/ijms21144867

**Published:** 2020-07-09

**Authors:** Jerome Fajardo, Bruce K. Milthorpe, Jerran Santos

**Affiliations:** Advanced Tissue Engineering and Stem Cell Biology Group, School of Life Sciences, Faculty of Science, University of Technology Sydney, Sydney, NSW 2007, Australia; Jerome.Fajardo@gmail.com (J.F.); Bruce.Milthorpe@uts.edu.au (B.K.M.)

**Keywords:** mesenchymal stem cells, neural, differentiation, phosphodiesterase inhibitor, IBMX, neurite, dendrites

## Abstract

Stem cells are highly important in biology due to their unique innate ability to self-renew and differentiate into other specialised cells. In a neurological context, treating major injuries such as traumatic brain injury, spinal cord injury and stroke is a strong basis for research in this area. Mesenchymal stem cells (MSC) are a strong candidate because of their accessibility, compatibility if autologous, high yield and multipotency with a potential to generate neural cells. With the use of small-molecule chemicals, the neural induction of stem cells may occur within minutes or hours. Isobutylmethyl xanthine (IBMX) has been widely used in cocktails to induce neural differentiation. However, the key molecular mechanisms it instigates in the process are largely unknown. In this study we showed that IBMX-treated mesenchymal stem cells induced differentiation within 24 h with the unique expression of several key proteins such as Adapter protein crk, hypoxanthine-guanine phosphoribosyltransferase, DNA topoisomerase 2-beta and Cell division protein kinase 5 (CDK5), vital in linking signalling pathways. Furthermore, the increased expression of basic fibroblast growth factor in treated cells promotes phosphatidylinositol 3-kinase (PI3K), mitogen-activated protein kinase (MAPK) cascades and GTPase–Hras interactions. Bioinformatic and pathway analyses revealed upregulation in expression and an increase in the number of proteins with biological ontologies related to neural development and substructure formation. These findings enhance the understanding of the utility of IBMX in MSC neural differentiation and its involvement in neurite substructure development.

## 1. Introduction

Stem cells are highly important in biology due to their unique innate ability to self-renew and differentiate into other specialised cells. Their potential in medical applications is rapidly growing in the areas of cellular and tissue repair and regeneration, and they also have the potential to treat and understand diseases. Currently, autologous stem cell therapy is limited to relatively simple clinical applications with minimal manipulation such as bone marrow transplants using adult mesenchymal stem cells (MSCs) [1]. MSCs can be found in adult mesodermal lineage tissue such as bone marrow [2] and adipose tissue [3]. Adipose-derived stem cells (ADSCs) have substantial advantages in their usage for regenerative medicine. Like other stem cells, they are multipotent in vitro and have challenged the dogma of lineage-only differentiation, showing potential in adipogenic, chondrogenic, osteogenic, myogenic and neurogenic differentiation capabilities [4,5,6]. In a neurological context, regenerative cellular treatments for major injuries are a strong basis for research in this area. These include traumatic brain injury (TBI) [7], spinal cord injury [8], and stroke [9]. Neural tissue alone has a limited capacity to regenerate [10], but stem cells have the broad potential to regenerate lost or impaired tissue function from injury [11].

In neurological disorders such as TBI, spinal cord injury and stroke, glial scarring occurs [12,13,14]. This can cause complications as glial scarring is a physical and chemical barrier created by the activation of astrocytes that surround the lesion core and wall off intact neurons [15]. It has the beneficial effect of sealing the injured tissue and preventing the spread of damage [15,16] by protecting damaged neural tissue [17], preventing a massive inflammatory response [18], and re-establishing the blood–brain barrier [19]. However, it has the detrimental effect of preventing axonal outgrowth [20], causing reduction in regeneration [18,19]. All these effects start to form within hours of the initial injury [21]. As such, there may be a limited time window to act and administer effective regenerative treatment.

A rapid interventional cellular therapy that can limit secondary injury by modulating the local tissue trauma environment as well as increasing function and neural repair is needed. There is interest in the use of small-molecule chemicals as inducers of neural differentiation in conjunction with MSCs. Small molecules are particularly useful because they have compelling advantages: they are easy to manufacture, administer and store; their effects are dose-dependent, specific and rapid; and the current regulatory environment supports their development and discovery as therapeutics [22].

Several chemicals have been cited in the literature and included as parts of chemical cocktails that reportedly cause neural induction, which may include small-molecule chemicals, growth factors, hormones or other proteins, [23,24,25,26,27] such as those containing indomethacin, insulin and isobutylmethylxanthine (IBMX) [28]; and those containing brain-derived neurotrophic factor (BDNF) and retinoic acid [29]. The previous use of small-molecule chemicals such as β-mercaptoethanol (BME), butylated hydroxyanisole (BHA), valproic acid (VA) and dimethyl-sulfoxide (DMSO) produced differentiated cells within a far shorter time than conventional methods, sometimes within minutes or hours [23]. This favoured property could allow drug and cellular intervention to be administered in a neural deficit before the onset of secondary injuries such as glial scarring, thus improving the regenerative process.

As part of cocktails, they are known inducers of differentiation, but the characterisation of each of the chemicals in terms of their individual contribution to the differentiation process has often not been well defined, often limited to staining for relatively few neural surface markers. Perhaps with the expectation that these small-molecule chemicals alone will probably not induce full differentiation. The use of neural induction media containing other less toxic small-molecule chemicals such as IBMX has been suggested to be possibly important in activating pathways vital to the differentiation process. It is thought to be a crucial instigator to modulating key molecular mechanisms thus driving downstream differentiation cues and the development of neural substructures.

IBMX is a non-competitive selective phosphodiesterase inhibitor [30] which increases intracellular cyclic adenosine monophosphate (cAMP) and inhibits leukotriene synthesis [31], an inflammatory mediator; it also inhibits tumour necrosis factor alpha (TNFα) synthesis, activates protein kinase A, and reduces innate immunity and inflammation [31]. When used in combination with dopamine, fibroblast growth factor (FGF), forskolin and 1, 12-O-tetradecanoylphorbol-13-acetate (TPA), it induces the expression of the dopaminergic neuron marker tyrosine hydroxylase in neurons derived from an embryonic carcinomal stem cell line (NT2) [32]. When used along with dibutyryl cyclic adenosine monophosphate (dbcAMP), which increases the intracellular concentration of cAMP, 25% of human MSCs differentiated into neuron-like cells expressing vimentin and NSE in culture after 6 days [33]. It is also known to induce neural differentiation from human umbilical cord blood-derived MSCs [34] and to promote the differentiation of rodent neural progenitor cells (NPCs) into functional neurons in vitro with 50% of differentiated cells firing action potentials [35].

The aim of this study is to investigate the effect of IBMX treatment in MSC differentiation to a neural-like lineage through phenotypic, proteomic and cytokine expression changes expounding on the molecular mechanisms involved.

## 2. Results

### 2.1. Microscopy and Morphological Changes in IBMX-Treated MSCs

**Figure 1:**
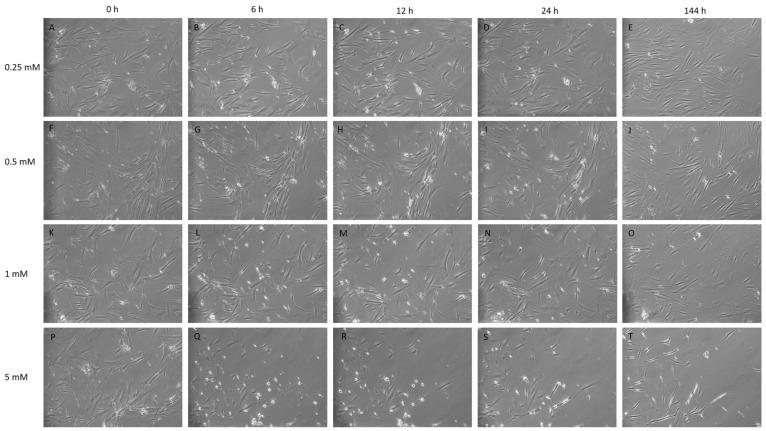
Isobutylmethyl xanthine (IBMX) treatment of mesenchymal stem cells (MSCs) at 10× magnification over time displaying cells in treated concentrations of 0.25, 0.5, 1 and 5 mM. (**A**–**E**,**F**–**J**): The lower concentrations 0.25 and 0.5 mM display tangible morphological changes such as cell narrowing, elongation and neurite-like structures between tge timepoints 12 h and 24 h. (**K**–**O**): 1 mM shows similar traits being displayed by the 6 h time point with progressively more defined features and neurite-like structure formation over time. (**P**–**T**): The 5 mM treatment shows a high loss of cells by detachment; cells acquire strong morphological changes by 6 h with the rounding of cells, with high light refraction and extensions linking between cells; the cell morphology by 144 h displays a dense elongated cell with a bipolar or tripolar body (**T**).

The chemical treatment of MSCs with IBMX was performed with the intent to observe phenotypic and morphological features congruent with neural cell types. Cultures were maintained at sub-confluency prior to chemical treatment. Typically, MSCs display large, flat, spindle-shaped or fibroblast-like morphology, while all cells post treatment show morphological changes, through all time points and treatment concentrations as seen in the Figure 1 panel. Cells undergoing differentiation display a neural-like morphology, exhibiting highly light-refractive rounded cells that are bipolar or multipolar in appearance with membranous, process-like extensions that resemble neurites reaching between cells (Figure 1).

Post-IBMX treatment cells showed the greatest phenotypic changes between 0 and 12 h, with cells having distinct neuronal features, including the rounding of cell bodies and the appearance of process-like extensions, appearing at later timepoints, from 24 h, for lower concentrations at 0.25 to 0.5 mM, and earlier, from 1–12 h, at higher concentrations, at 5 mM.

Typically, few morphological changes occurred at the lower concentration of 0.25 mM at early timepoints, as shown in Figure 1C. Later timepoints for this treatment exhibited slender cells with extensions. Treatments of 0.5 and 1 mM presented a steady cell number with minimal losses and far more distinct morphological changes accrued over time, with cells progressively becoming more defined with processes and extensions reaching between cells. Neurite-like structures appeared within 12 h of treatment at 1 mM becoming more defined at 24 h (Figure 1H,M) and maintaining this morphology through later daily images (data not shown) with no further media changes until the final timepoint at 144 h. At early timepoints for the 5 mM treatment, there were a greater number of cells with a differentiated neuronal appearance but also a noticeable reduction in confluency, indicating a greater amount of cell loss. This is shown in Figure 1Q,R. Phenotypic changes tended to appear as sporadic pockets or clusters of cells. As a result, proteomic analysis was performed on cells treated at 0.25, 0.5 and 1 mM and the additional treatment of 2.5 mM, up to the 24 h timepoint.

### 2.2. Cell Counts and Cytotoxicity Assay

**Figure 2:**
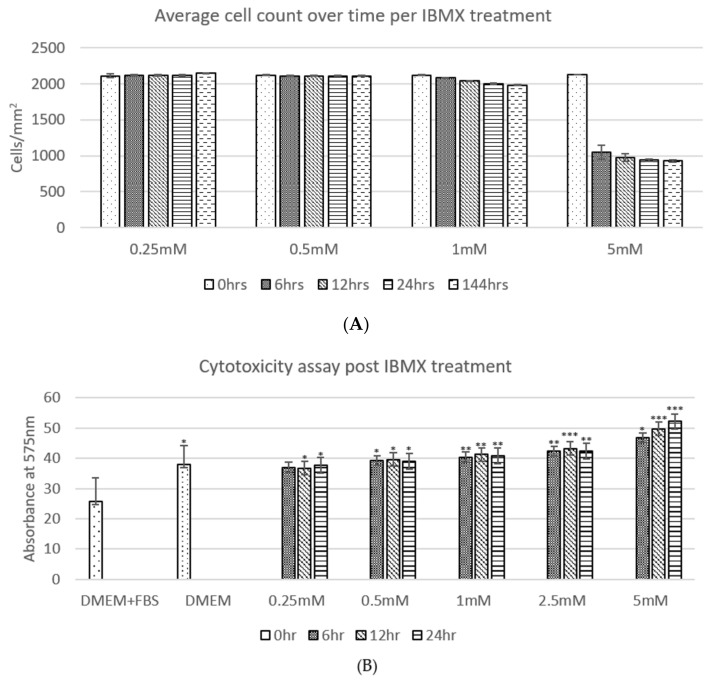
(**A**) shows the average cell count over time per IBMX treatment: 0.25–1 mM show relatively minimal changes with no statistical significance using the Student’s *t*-test, while the 5 mM treatment shows a loss of up to 50% of cells by detachment. (**B**) shows the cytotoxicity assay post IBMX treatment displaying statistical significance relative to each sample compared to DMEM and FBS using a one-way Student’s *t*-test where the *p*-value is presented as * < 0.05; ** < 0.01 and *** < 0.005. The two graphs display a relative correlation between the measured cytotoxicity levels and average cell numbers.

The average cell counts (Figure 2A) of ADSCs post IBMX treatment over time shows a clear trend, where the ADSC cell population per square centimetre across technical replicates remains relatively unchanged in 0.25 and 0.5 mM treatments over the 24 h period and up to 144 h. The 1 mM treatment shows a marginal decrease in cell number; however, it has no statistical significance over time. The 5 mM treatment shows a stark difference in cell number decrease between 0 and 6 h post treatment with at least 50% of the cell population no longer adhering to the culture dish surface. The remaining population from 6 h to 144 h then remained relatively steady with minor detachment at the remaining timepoints. Due to the sizeable loss of cells in the 5 mM treatment between 0 and 6 h, a further concentration treatment was added to the regime trials at 2.5 mM tested in the following cytotoxicity assay as well. To assess the cell detachment to cytotoxicity imposed by the increasing IBMX treatment a cytotoxicity assay was completed (Figure 2B). The relative trends observed across the board tend to correlate cytotoxicity to average cell numbers. IBMX treatments of 0.25 and 0.5 mM show an equivalent level to that of serum starved cells and marginally higher ones for 1 and 2.5 mM. The final treatment of 5 mM exhibits a steady increase over time that inversely correlates to the decrease in cell numbers, therefore suggesting that the cell number decrease is related to cytotoxicity by high concentrations of IBMX; however, half its concentration (2.5 mM) remains safe.

### 2.3. Mass Spectrometry of Proteins from IBMX-Treated MSCs

Each IBMX treatment of cells was completed in biological triplicate. Each of these samples were then analysed by mass spectrometry in technical triplicate, which allowed for high stringency and robust analysis across 14 different treatment timepoints in 40 different replicates. The mass spectrometry data acquired identified, at the 95% confidence cutoff, a total of 1621 unique proteins. The identified proteins were analysed through ClueGO (version 2.5.7) identifying the biological processes’ ontology definitions. There were 172 gene ontology biological process categories of proteins identified in this study that directly relate to neural differentiation, development, regulation of cascades, migration, extensions and the formation of neural substructures (Table S1).

**Figure 3:**
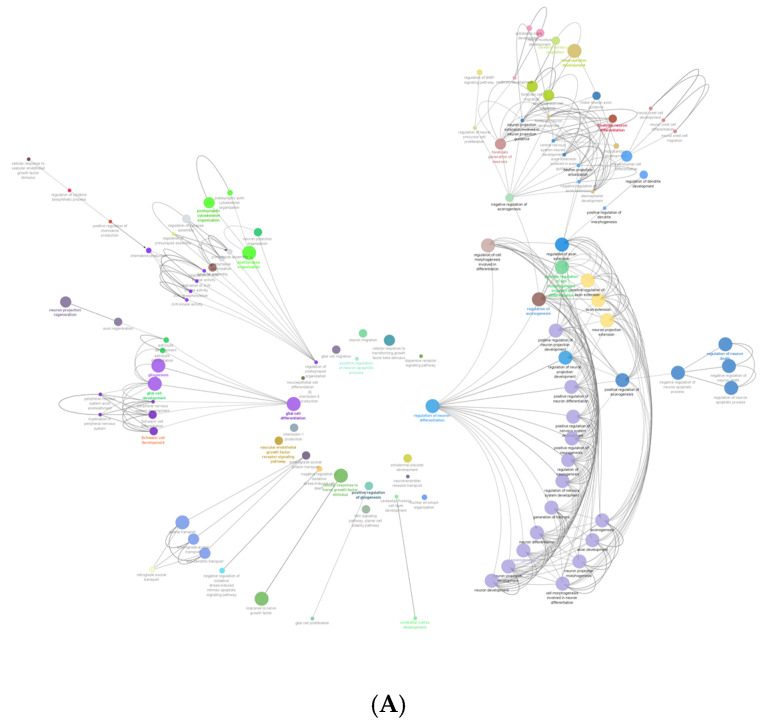

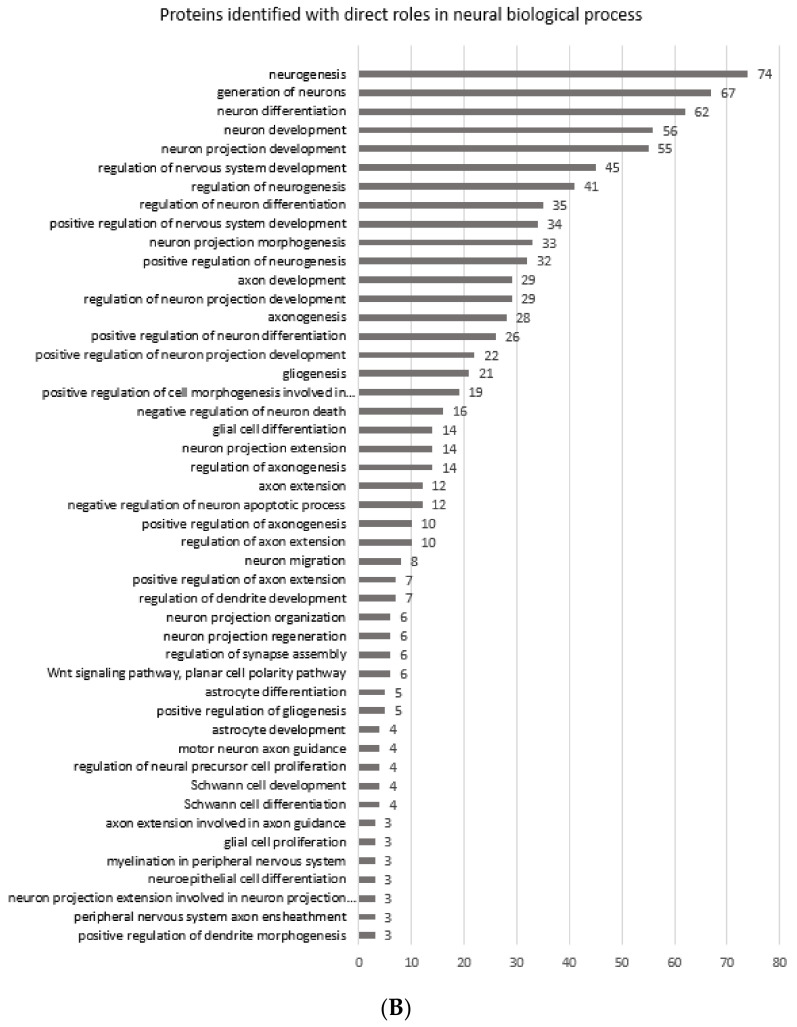
(**A**) shows the gene ontology radial Cytoscape network of the proteins identified in the biological processes with neural associations. (**B**) shows the refined number ontologies of proteins identified with direct roles in a neural biological process.

Figure 3A shows the gene ontology radial Cytoscape network of the proteins identified in the biological processes with neural associations in this study. The network presents the ClueGo database-annotated interactions between the biological processes, revealing the links between cascades, pathways and the expressed proteins involved in neural development. Figure 3B breaks down the numbers of proteins within each of the presented ontologies within the network graph. Figure 3 shows the parent biological process categories directly related to neural differentiation, development, and the formation of neural substructures. The top ten categories with the highest number of proteins were involved in neuron development. There were up to 74 proteins involved in neurogenesis. All subsequent categories had numerous proteins involved in each process with a lowest number of three proteins identified in the positive regulation of dendrite morphogenesis.

The analysis of protein expression by relative quantitation was completed where the presence and comparative ratios of proteins expressed in the sample compared to the Dulbecco’s Modified Eagle’s Medium with Ham’s F-12 (DMEM) and Fetal Bovine Serum (FBS) control proteins identified were based on the significance method PEAKSQ with a fold change of more than or equal to 2, with at least one unique peptide. Their relative expression to the basal ADSCs in DMEM and FBS control was expressed as a relative ratio number between 0 and 64, i.e., as a ratio where a number less than 1 signified downregulation and any number above 1 signified upregulation. Proteins uniquely expressed post IBMX treatment do not have a ratio due to the absence of the protein in the denominator DMEM and FBS control; these are presented in Table 1. In the IBMX analysis, 48 proteins were identified as having probable biological significance as markers of differentiation as part of the proteomic analysis. These were identified by either their presence in treatment replicates and their absence in both the FBS control and the DMEM control; or their relative similar expression in both controls (expression ratio between 0 and 2 for the DMEM control) in addition to their fold change in expression from the FBS control being greater than 2.00 in at least six, or half, of the replicate treatments (Table 1). These proteins were then screened for at least three relevant search terms related to neural/neural differentiation in their gene ontology of biological processes. A list of proteins with the highest number of associated biological processes and strongest relevance to neural differentiation and development is given in Table 2.

For proteins identified in this analysis, the distribution of high ratios of expression was counted and is presented in Table 3. For each protein that appeared in the FBS control and was upregulated in IBMX-treated MSCs, the number of times the expression levels were above 2.00 and 5.00 was counted for each treatment condition. For the proteins that were uniquely expressed only in IBMX-treated MSCs and not in the basal MSCs, no ratio is able to be calculated, thus the presence of the unique protein is designated by a tick (✓) in Table 3. If the same protein was not detected in other treatments it is designated by a cross (✕). As such, the number of occurrences of uniquely expressed proteins for each treatment condition was counted.

It was quantitatively determined that the most frequent neural inductive activity or change occurred proteomically at these treatment conditions. It was consequently found that the highest counts of the cumulative data occurred at 0.5, 1 and 2.5 mM at 6 h. A similar analysis utilising all the proteins that were upregulated in the same described way, without excluding those that were not neurally related as described on UniProt, consisted of 202 proteins total in the analysis.

### 2.4. Cytokine and Chemokine Expression Changes in MSCs Post IBMX Treatment

**Figure 4:**
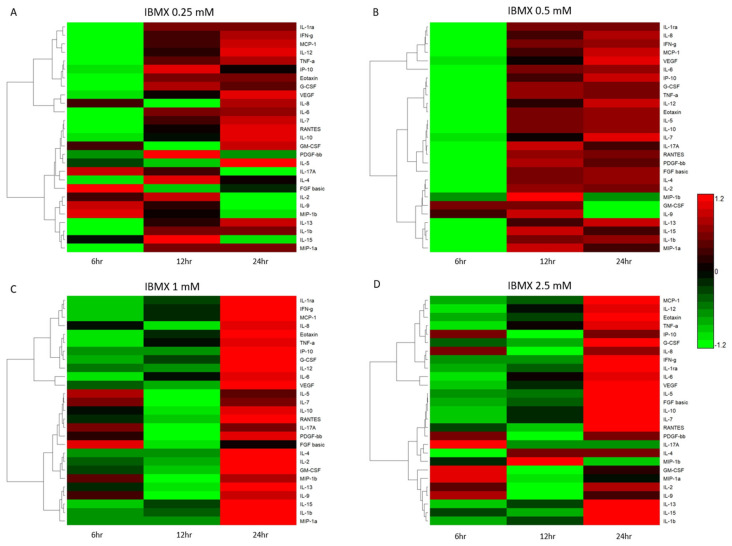
Bioplex quantified cytokines over time heatmap representations of IBMX–treated MSCs: (**A**) 0.25 mM, (**B**) 0.5 mM, (**C**) 1 mM and (**D**) 2.5 mM.

Temporal cytokine expression levels post IBMX treatment have a biological implication on the cells they are secreted from. Figure 4 shows the varied cytokine concentrations across the four treatments over time. Further clarifying their variation is the Euclidean clustering dendrogram of the 27 measured cytokines. Each treatment displays similarities as well as differences in the cluster dynamics. IBMX treatments of 0.25 and 0.5 mM tend to have similar overall clustering trends with variation in quantitation. Treatment at 2.5 mM presents unique clustering patterns as cytokine levels change with an increase in IBMX concentration. Not surprisingly the 1 mM treatment shows cluster similarities between the 0.5 and 2.5 mM treatments; however, the cytokine concentration levels are closer at the 24 h timepoints between 1 and 2.5 mM. Examining the dendrogram clustering profiles, Figure 4A,B has the same pattern for MIP-1a, IL-1b, IL-15 and IL-13, and whilst their group clustering is the same between 0.25 and 0.5 mM IBMX treatments, the relative expression varies. Complementary to this trend, there are variations in the molecules clustering in the remaining two treatments. In the 1 mM treatment the IL-9 levels show inclusion in the group cluster whereas in the 2.5 mM treatment the IL-2 and IL-9 levels are closer to that of IL-1b, IL-15 and IL-13, displacing MIP-1a to a proximal cluster (Figure 4C,D). Another two highly expressed molecules alter their clustering patterns over time and treatment. IL-6 and VEGF are distally related in clusters in the 0.25 and 0.5 mM treatments; however, in the 1 and 2.5 mM treatments, they are clustered together in a unique grouping and markedly higher levels than the preceding treatments.

## 3. Discussion

This study investigated the neurogenic inductive potential of the small-molecule chemical and phosphodiesterase inhibitor IBMX, with its treatment of MSCs in various concentrations over time. The resultant cells were examined by live cell microscopy, cytotoxicity assay, secretory cytokine assays, proteomic analysis and bioinformatic interrogation by systems biology. To derive a greater understanding of the range of molecular and phenotypic changes involved in neural substructure development that occurred over time during treatment. The proteins identified by the analytical process reveal many common neural related processes and functions between them. It is also known that in vivo neurons are generated from neuronal progenitor cells (NPC) and start a long maturation process with many steps including neurite extension, axon elongation, dendrite formation and neuronal migration [36]. There is a prevalence in the literature of these processes and their relation to the maturation of neurons. The subsequent sections explore the roles of the identified proteins, principally due to the relation to neural induction with IBMX.

### 3.1. Neurite Development and Protein Signalling Mechanisms

There is a recurring frequency in the identified proteins related to biological processes involving neurites. Neurites are projections from the cell body of a neuron, which can be either an axon or a dendrite. Proper dendritic and axonal morphogenesis and neurite outgrowth are important for neuronal function, synaptic formation and neuronal maturation [37,38]. Neurite growth requires complex processes for cytoskeleton formation, which is driven by extracellular and intracellular signalling. One of the important parts of the cytoskeleton is the microtubules, consisting of heterodimers of tubulin [39]. Microtubules localised at the end of a neurite, capable of shortening and joining, are called the growth cone [40]. Molecular interactions between the uniquely expressed proteins Adapter protein crk, hypoxanthine-guanine phosphoribosyltransferase (HPRT), DNA topoisomerase 2-beta (Topo IIβ) and CDK5 in the treated MSCs analysed proteome have significant biological implications for the substructure formation and development cues that drive them.

The identification of Adapter protein crk expressed in the IBMX-treated ADSCs was particularly intriguing, since Adapter molecule crk regulates cell adhesion, spreading and migration and neurite outgrowth. A study into the introduction of this protein in PC12 cells induced neuronal differentiation with neurite formation. It was also found that antibodies against either the src homology region 2 (SH2) region (a region required for signalling) or GTPase Hras suppressed neuronal differentiation. This suggests that the SH2 region of Adapter protein crk generates a signal for neurite differentiation through the activation of GTPase Hras [41]. The neuronal guidance cues involved through Adapter protein crk also link to the Reelin signalling cascade that controls neuronal migration and cellular adhesion during neuronal development [42]. A study by Matsuki et al. showed that a reduced expression of Crk family proteins can block the reelin-induced formation of dendrites [43]. Interestingly, Reelin expression is modulated by RAS-PI3K signalling, which also promotes the previously mentioned GTPase HRAS [44], both having significant expression fold increases in this study. PI3K signalling has been shown to play a role in neurite outgrowth in a study of neuronal cells treated with atorvastatin [45]. A further implication of the effect of IBMX was the modulation in the expression of cytokines. A rise in IL-2 levels increases the regulation of Crk family proteins’ interactions with STAT5 [46], STAT5 being integral to neurite outgrowth [47]. IL-2 has also been previously implicated in sympathetic neurite outgrowth [48]. Complementary to this, the increasing levels of the closely trending Bioplex cluster of IL-6 and VEGF in this study also have secondary and tertiary interactions with Crk family proteins, supporting neurite outgrowth and neuronal assistance supposition [49,50].

Similarly, inactive tyrosine-protein kinase 7 has implications for neurite development and is non-canonically involved in the Wnt pathway that is synonymous with cellular reorganisation, cell elongation and migration during differentiation. A study performing a gene “knockdown” screening cell array using a tyrosine kinase-specific siRNA library used an analysis indexing neurite outgrowth. In the four tested subarrays, neurite extension was inhibited in SH-SY5Y neuroblastoma cells, and was found to be inhibited in a statistically significant way in at least two screening tests as compared to random siRNA controls for the inactive tyrosine-protein kinase 7 [51]. As well as this, the expression of Cadherin-2 in the IBMX-treated MSCs further supports the activation of neurite outgrowth. In this case, an engineered Cadherin-2 substrate was used in a study of the treatment of mouse neural progenitor cells, which led to the increase of neurite outgrowth and a faster growth rate in the cells. This implies that the expression of these proteins, in IBMX-treated ADSCs, functions similarly to that in developing neural cells by activating the Wnt and related pathways that guide structural development in differentiation in treated ADSCs.

The expression of the enzyme hypoxanthine-guanine phosphoribosyltransferase (HPRT) in the treated samples has an important biological implication. It is involved in the purine nucleotide salvage pathway and plays an intermediary role in linking the metabolic process and neuron differentiation in central nervous system development. The expression of this protein can be thought of as a direct response to the presence of low concentrations of IBMX at early treatment timepoints, in which a phophoribosyl group is directly added to IBMX, possibly altering its structure to make it closer to an adenosine-like shape; thus, IBMX would then enter the nucleotide salvage pathway. Furthermore, HPRT also plays a role in the Wnt pathway which, as previously mentioned, is involved in neurite outgrowth regulation. Supporting this was a study by Guibinga et al. of HPRT-deficient neurons, differentiated from pluripotent NT2 cells, showing wild-type electrophysiological properties, but with a significant reduction in neurite outgrowth during differentiation [52]. This signifies that HPRT has an important role in the neurite outgrowth function in neural cell development.

Another enzyme abundantly expressed post chemical treatment with biological implications for neurite formation is DNA topoisomerase 2-beta (Topo IIβ). In Topo IIβ knockout mice, there was a significant loss of dopaminergic neurons and a lack of neurites. Topo IIβ suppression via ICRF-193, a specific TOP II antagonist, or via Top IIβ siRNA in the primary cultures of ventral mesencephalic neurons resulted in growth cone collapse and neurite shortening [53]. Not surprisingly, human MSCs that were neurally differentiated and Topo IIβ-silenced lost their morphology, becoming flattened and enlarged, along with reduction in both neural differentiation efficiency and neurite length. In contrast, Topo IIβ overexpression caused the human MSCs to exhibit neural cell morphology, characterised by longer neurites [54]. A final protein within the upper echelon with an important role identified in the datasets of treated MSCs in relation to neurite development was CDK5 regulatory subunit-associated protein 2 (CDK5RAP2). It binds to p25nck5a, a subunit of CDK5 (also known as neuronal CDC2-like kinase [55]). CDK5 plays an important dual role in controlling neurite outgrowth and axon and dendrite development, and is involved in axon elongation and dendrite arborisation. The presence of CDK5 reinforces the biological value of all the aforementioned proteins involved in neurite development and outgrowth that naturally act in the development of axons and dendrites.

### 3.2. Protein–Protein Interaction Signalling Involved in Axon Development

An axon is a long neuronal process that conducts information from the cell body to the nerve terminal [56]. The function of the axon is to transmit information to different neurons, muscles and glands. Protein–protein signalling plays a vital role in the development of axons and neuronal maturation, and it is important to explore the signalling roles in the differentiation of MSCs in response to IBMX treatment. Receptor molecules are the gatekeepers that receive cellular signals from extracellular small molecules like IBMX outside the cell, or from secondary messengers within the cell in response to other interactions. Inactive tyrosine-protein kinase 7 and Neuropilin-2 (NRP2) are two receptor proteins identified in the IBMX-treated MSCs that have very closely related functions. Inactive tyrosine-protein kinase 7 forms complexes with the plexin family of proteins, which are transmembrane molecules that transduce signals from ligands of the semaphorin family [57] (146). NRP2 shows high affinity binding to class-3 semaphorins [57,58], where neuropilins are recruited by A-type plexins as the ligand binding subunit for class-3 semaphorins, and other plexins such as plexin D1 are known to bind directly to semaphorins [59]. Neuropilin and plexin receptors are known to receive local semaphorin signalling to affect axon growth cones and impede axonal growth in the direction of the received signal [60]. While most studies suggest that plexins are the only molecules capable of triggering an intracellular signal, several reports indicate that neuropilins can transduce a signal independently, such that neuropilins appear to function as more than a simple stabilising component for the semaphorin/plexin complex [61]. Semaphorins have also been described to be involved in cytoskeleton reorganisation, affecting motility, cell–to-cell interaction and cell shape [57]. This implies some redundancy in the roles played by the complement of selected proteins in axonal directional growth, and thus the importance in the downstream processes that lead to the differentiation of MSCs.

Signalling proteins that have several types of interactions dependent on their subcellular location and expression levels can act as an intermediary to coordinate multiple interactions and signalling mechanisms. The previously mentioned GTPase HRas is an interesting example of a protein expressed, in IBMX-treated MSCs, with multifunctionality in the differentiation process. It is involved in the development of neuronal polarity, which is caused when one of the minor neurites undergoes rapid outgrowth and becomes an axon. This is regulated by signalling elements related to PI3K, which stimulate microtubule and actin re-organisation. GTPase HRas is an upstream regulator of PI3K which increases at the beginning of axonal growth cone creation, but appears to be transported out of the remaining neurites in order to limit formation to one axon [62], which is an interesting function in the control of proper and regulated axonal growth, especially in the context of the neuronal differentiation of MSCs. The pleiotropic signalling molecules measured in the bioplex assay have innumerable interactions. However, growth factors stand out from this group. FGF-basic or bFGF is a growth factor and member of the fibroblast growth factor family, and generally regulates differentiation, migration and cell growth and survival during regeneration and development [63]. It is known to have a role in stem cell biology in the maintenance of stemness and differentiation control [64]. bFGF functions as a highly expressed neurotrophic factor and differentiation factor in the central nervous system. Specifically, in cases where bFGF is exposed to MSCs it has proven differentiating effects. When treated with bFGF, mouse MSCs express voltage-dependent channels, functional dopamine receptors and neuron-specific proteins, with neuron-like K+ outward currents [65]. When canine MSCs are treated with bFGF alone they express neuron-specific mRNAs for Neuron Specific Enolase (NSE), Neurofilament-L (NF-L), and Microtubule Associated Protein 2(MAP2), and proteins for NSE and NF-L, and exhibit neuron-like morphology. A steep increase in intracellular Ca^2+^ concentrations when invoked with L-glutamate and potassium chloride has also been observed, suggesting that this induced differentiation into glutamate and voltage-responsive neuron-like cells [66]. When human MSCs were treated with bFGF this contributed to their differentiation into functional neuron-like cells where they expressed RNAs and proteins that were neuron-specific, as well as being dopamine-secreting and voltage-responsive in neuron-like ways [67,68]. The increased expression of bFGF at early timepoints of IBMX-treated MSCs presents positive biological implications for neural differentiation. As bFGF is also involved in many signalling pathways including the PI3K/Akt, MAPK/ERK kinase (MEK) and the extracellular signal-regulated kinase (ERK) pathway. In mouse MSCs, bFGF is important in neuronal differentiation via the MEK/ERK pathway [69]. Via the PI3K/Akt pathway, bFGF mediates cell survival neuronal differentiation, primary NSCs, ESCs and embryonic carcinoma cell lines (P19 cells) [70]; and in PC12 cells it suppresses endoplasmic reticulum stress-induced apoptosis [71]. The PI3K pathway and the endoplasmic reticulum stress response appear to connect bFGF with GTPase Hras and GRP78, as described earlier, which adds credibility to the importance of these proteins in neural differentiation.

The outcome for a signal or cascade is the proteins expressed or inhibited downstream in a pathway. The result can lead to a phenotypic change, in this case the expression of structural proteins driving structural and functional changes. Neurofilaments are important proteins that are required for axon radial growth and the NF-L subunits in particular are important for this [72]. Reduced electrical conduction velocity concomitant with decreased axonal calibre (diameter) has been observed in knockout mice that lack the gene for NF-L [73] and in a nonsense mutation in the gene for NF-L in mutant Japanese quail [74], which outlines the role of this protein as not just as an early marker in neuronal cell differentiation, or just to regulate axonal size. It also has importance in the speed of impulses generated by the neuron cell body, all of which is important in signifying that there are indications that the treatment of MSCs with IBMX induces some partiality to neuronal differentiation with the correct molecular cues and signalling.

### 3.3. Conclusions

In this study we presented the treatment of MSCs with a range of concentrations of IBMX over time. The subsequent changes were profiled by proteomics, bioplex assay, gene ontology and signalling pathway analysis. The results obtained indicate that the phenotypic changes induced by the small-molecule treatment were in favour of promoting the molecular mechanisms and signalling pathways in neural differentiation substructure formation. IBMX-treated MSCs’ morphological and phenotypic changes were observed with increasingly differentiated neuronal features over increasing concentrations and periods of time. Most promising was the observed marked increase in bFGF in response to chemical treatment, as bFGF is a known functional neuronal differentiator of MSCs. The relation of bFGF to the PI3K pathways and the endoplasmic reticulum stress response appear to connect bFGF with GTPase Hras and GRP78 interactions. Most importantly, the uniquely expressed proteins Adapter protein crk, HPRT, Topo IIβ and CDK5 in the treated cells linked numerous other unique and shared upregulated proteins across the interaction network pathway. This displayed a sizeable increase in proteins with biological ontologies related to neural development and substructure formation. Many identified proteins may prove useful as markers in future characterisation studies. Further investigation may lead to identifying optimal neural differentiation states, which may be more desirable and clinically important as the differentiating cells may orientate and mature terminally in the presence of surrounding chemotactic and paracrine signals. This study may assist in establishing opportunities to design specific chemical therapeutics which would enable the treatment of neural injuries and diseases.

## 4. Materials and Methods

### 4.1. Cell Culture

ADSCs used for the experiments were previously isolated using methods adapted from Bunnell et al. [75] and Santos et al. [76] under University of Technology Sydney Human Research Ethics approval Santos-2013000437 (Approval on 02/07/2013). All donor participants volunteered through informed consent for waste lipoaspirate donation as per ethics guidelines and were de-identified for research purposes. Cells were cryo-stored in 90% fetal bovine serum (FBS)/10% DMSO and subsequently revived in 90% Dulbecco’s Modified Eagle’s Medium with Ham’s F-12 and GlutaMAX (DMEM/F12 GlutaMAX, Gibco, Life Technologies, Carlsbad, CA, USA) with 10% FBS (Gibco). Cells which were passaged 6–9 times were grown in 80% DMEM/F12 + GlutaMAX and 20% FBS (Gibco, Life Technologies, Carlsbad, CA, USA) at 37 °C and 5% CO_2_ until at least 80% confluent, then were dissociated from the surface with TrypLE Express (Gibco, Life Technologies, Carlsbad, CA, USA) and re-seeded at 20% confluency on 6-well plates to grow to at least 80% confluency. Each experimental condition was made in triplicate.

### 4.2. Cell Neural Induction

Induction was performed with isobutylmethyl xanthine (IBMX) where cells were cultured in serum-free media for 12 h with a pre-induction media containing DMEM/F12 + GlutaMAX and 10% dosage of the test chemical only (0.025, 0.050, 0.1, 0.25 or 0.5 mM IBMX) for serum starvation and pre-induction treatment. Media was then replaced with serum-free neural induction media containing DMEM/F12 + GlutaMAX and the designated final dosage of IBMX (0.25, 0.5, 1, 2.5 or 5 mM IBMX). The ranges chosen were based on previous studies which exposed cells to similar chemical ranges [34,35]. Non-treated cells in standard basal media and a serum starved with no chemical controls were also completed. Images were captured at 10× magnification on an Evos XL (Life Technologies Thermofischer, Carlsbad, CA, USA) hourly up to 24 h then every day up to 6 days.

### 4.3. Cell Harvesting and Sample Preparation

The cells were harvested for proteomic analysis as per Santos et al. [27] for liquid chromatography-tandem mass spectrometry (LC-MS/MS), at time points of 0, 3, 6 and 24 h post treatment, were completed in biological and technical triplicates. Culture media was collected from each well in 2 mL Eppendorf tubes and stored at −80 °C for later Bioplex and toxicity assays. Cells were rinsed twice in 5 mL of 1× phosphate buffered saline (PBS, Merck KGaA, Darmstadt, Germany) for 5 min each at 37 °C and aspirated. Cells were then scraped into 1 mL of 1× PBS using a cell scraper (Sarstedt, Numbrecht, Germany) and the liberated cells were collected into an Eppendorf tube and centrifuged at 4000× *g* for 10 min. The supernatant was then discarded, and the cell pellets were stored at −80 °C until processing.

### 4.4. Cell Lysate Protein Extraction Sample Preparation

The cell pellets were resuspended in 100 µL 8 M urea (Merck KGaA, Darmstadt, Germany) and 100 mM ammonium bicarbonate (Merck KGaA, Darmstadt, Germany), and sonicated for 10 min at 50% power at three 10 s intervals. The samples were then heated to 95 °C on a heat block for 10 min, then centrifuged for 1 min at 5000× *g*. The solution was then reduced and alkylated by adding a final concentration of 10 mM tributyl-phosphate (TBP, Merck KGaA, Darmstadt, Germany) and 20 mM acrylamide (Merck KGaA, Darmstadt, Germany), then vortexed and spun down on a mini-centrifuge (Qik Spin QS7000 Edwards Instruments, Elkhorn, WI, USA) at 2000× *g* for 2 s. The samples were incubated for 90 min at room temperature then quenched with a final concentration of 50 mM dithiothreitol (DTT, Merck KGaA, Darmstadt, Germany) and again vortexed and spun down on a mini-centrifuge at 2000× *g* for 2 s. The samples were then diluted 1:8 in 100 mM ammonium bicarbonate. We then added 0.5 µg of trypsin to digest at 37 °C for a minimum of 12 h. The samples were then desalted using Stop and Go Extraction (STAGE) tips solid phase extraction columns. The peptide concentration was determined using the Pierce quantitative colorimetric peptide assay (Thermofisher Scientific, Sydney, NSW, Australia) and prepared for LC-MS/MS analysis.

### 4.5. Liquid Chromatography-Tandem Mass Spectrometry

An Acquity M-class nanoLC system (Waters, Milford, MA, USA) was used, loading 5 µL of the sample (1 mg) at a rate of 15 mL/min for 3 min onto a nanoEase Symmetry C18 trapping column (180 mm × 20 mm). It was then washed onto a PicoFrit column (75 mm ID × 250 mm; New Objective, Woburn, MA, USA) packed with Magic C18AQ resin (Michrom Bioresources, Auburn, CA, USA). The column was then eluted of peptides into the Q Exactive Plus mass spectrometer (Thermofisher Scientific, NSW, Australia) using the following program: 5%–30% MS buffer B (98% acetonitrile + 0.2% formic acid) for 90 min, 30%–80% MS buffer B for 3 min, 80% MS buffer B for 2 min, 80%–5% for 3 min. The peptides that were eluted were ionised at 2000 V. A data-dependent MS/MS (dd-MS2) experiment was performed with a 350–1500 Da survey scan performed at a resolution of 70,000 *m*/*z* for peptides of charge state 2+ or higher with an automatic gain control (AGC) target of 3 × 106 and a 50 ms maximum injection time. The top 12 peptides were selectively fragmented in the higher-energy collisional dissociation (HCD) cell using a 1.4 *m*/*z* isolation window, an AGC target of 1 × 105 and a 100 ms maximum injection time. The fragments were scanned in the Orbitrap analyser at a resolution of 17,500 and the product ion fragment masses were measured over a 120–2000 Da mass range. The mass of the precursor peptide was then excluded for 30 s.

### 4.6. Mass Spectrometry and Protein Identification

The MS/MS data files were searched against the Human Proteome database and against common contaminants using Peaks Studio version 8.5 with the following parameter settings: fixed modifications: none; variable modifications: propionamide, oxidised methionine, deamidated asparagine; enzyme: semi-trypsin; number of allowed missed cleavages: 3; peptide mass tolerance: 30 ppm; MS/MS mass tolerance: 0.1 Da; charge state: 2+, 3+ and 4+. The search results were filtered to include peptides with a –log10P score (related to *p*-value) determined by the false discovery rate (FDR) of less than 1%, where the score indicated that decoy database search matches were less than 1% of total matches. Each condition was made up of the biological replicates that were treated at the same time, run in triplicate. The only exception was the FBS and DMEM controls, which were only grouped in duplicate, as the third samples were lost in a failed trial run to quantify protein load in the STAGE tip.

### 4.7. Data Analysis and Programming

A Python script was constructed to analyse and process the proteomics data. The code and annotations explaining this script are included in the Supplementary Materials. Proteins for this analysis were selected in two different ways: proteins that were present in the test samples but were not present in either control, or proteins that were at least upregulated by a ratio of 2 compared to the FBS control. These proteins were searched by unique accession number on UniprotKB (www.uniprot.org) and the corresponding biological processes were returned for each protein as an Excel spreadsheet.

The script did a number of things; by using the UniProtKB Excel spreadsheet, it returned the biological processes matching a search term for each protein, separated each term into a new row along with its protein, then proceeded to count how many times a protein occurred and how many times a biological process occurred for each search term. It created new sheets for each of these operations. The search terms included mostly partial word matches such as “astro” for astrocyte, “axo” for axon, “brain”, “cere” for cerebrum or cerebellum, “cort” for cortex or cortical, “cyto” for cytoskeletal, “dend” for dendrite, “diff” for differentiate, “dopa” for dopamine, “glia” for glial, “glutam” for glutamate, “neur” for neural or neurite, “nerv” for nerve or nervous, “prolif” for proliferate, “spina” for spinal, “stem” for stem cells, etc.

### 4.8. Cytokine and Chemokine Bioplex Analysis

Bioplex analysis was performed as per Santos et al. [27] with 500 μL aliquots collected at timepoints and controls as follows: DMEM control, starve, B27 control, 0, 3, 6 and 24 h. The assay was performed using Bioplex human 27-plex (M50-0KCAF0Y Bio-Rad Laboratories, Hercules, CA, USA). The data analysis was completed using DanteR software (DanteR version 1.0.0.10, R version 2.12.0, The R Foundation for Statistical Computing, Auckland, New Zealand) [77].

### 4.9. Resazurin Toxicity Assay

The assay was performed on cell supernatant to determine differences in cell stress levels caused by the different concentrations of chemical treatments made over a 24-h test period. The test detects the irreversible chemical reduction of resazurin to resorufin, which causes a change in colour from the blue reactant to the pink end-product. With increasing stress response from the cells, more reductive enzymes are excreted into the media, causing more resazurin to reduce into resorufin, which can be detected by an increase in absorbance. This was performed by adding 50 µL of test sample to 5 µL of alamar blue reagent (Invitrogen, Thermofischer, Carlsbad, CA, USA) in a 96-well plate, then incubating this for 2 h at 37 °C. Samples were performed in biological triplicate. Absorbance was read at 570 nm with 600 nm as the reference wavelength in a TECAN Infinity 200 plate reader (Tecan Group Ltd., Männedorf, Switzerland). Statistical significance was determined by the Student’s *t*-test for each sample relative to DMEM and FBS where the *p*-value is presented as * <0.05; ** <0.01 and *** < 0.005.

## Figures and Tables

**Table 1:** Fold change of upregulated proteins and relative ratios of IBMX-treated MSCs compared to basal MSCs in FBS control.

Protein Name	Controls		6 h				12 h				24 h			
	FBS	DMEM	0.25 mM	0.5 mM	1 mM	2.5 mM	0.25 mM	0.5 mM	1 mM	2.5 mM	0.25 mM	0.5 mM	1 mM	2.5 mM
60S ribosomal protein L27	1	0	2.74	4.03	2.96	3.87	9.41	5.82	1.92	3.73	6.85	8.17	7.06	1.8
Actin cytoplasmic 2	1	0.56	1.5	2.4	2.85	3.85	3.39	3.49	1.18	2.03	2.47	2.82	2.07	2.56
Alpha-actinin-1	1	1.63	7.52	13.23	15.81	11.59	13.35	13.27	5.33	6.04	3.98	7.36	8.54	8.42
Annexin A11	1	1.71	2.76	3.67	4.16	4.17	3.22	3.5	2.22	2.52	3.33	3.53	4.39	3.57
Annexin A6	1	0.59	4.87	10.98	7.18	10.95	5.84	8.98	4.61	4.5	7.64	6.88	3.62	6.46
Arginine and glutamate-rich protein 1	1	0.18	1.83	2.8	3.35	7.05	8.72	4.6	2.02	2.72	11.89	5.45	2.76	3.4
Calmodulin-like protein 3	1	0.79	3.6	6	6.58	9.68	14.65	5.64	4.63	7.64	13.29	18.13	14.24	6.66
Cation-dependent mannose-6-phosphate receptor	1	0	7.98	15.85	19.73	36.43	47.85	20.66	10.19	18.4	13	42.04	23.26	22
Chondroadherin-like protein (Fragment)	1	1.36	3.12	11.94	12.25	14.82	12.12	11.51	0.96	2	5.36	2.64	2.23	3.19
Collagen alpha-1 (XXIV) chain	1	0	2.02	6.81	8.26	7.18	15.14	2.51	0.97	3.69	23.32	9.39	7.28	3.26
DNA-directed RNA polymerase mitochondrial	1	1.55	4.29	5.48	6.29	9.69	8.54	3.79	2.58	4.69	4.92	6.04	4.45	4.4
Dolichyl-diphosphooligosaccharide-protein glycosyltransferase subunit STT3A	1	2	3.11	4.34	3.87	3.2	4.53	2.97	2.66	2.45	2.07	2.54	3.07	5.14
E3 SUMO-protein ligase RanBP2	1	1.97	2.43	3.35	6.07	4.71	9.47	3.23	3.47	4.08	3.02	13.41	5.18	5.18
EF-hand and coiled-coil domain-containing protein 1	1	1.02	1.9	3.69	3.54	3.82	3.09	7.06	2.15	2.23	1.48	2.34	1.78	2.09
Glucosidase 2 subunit beta	1	0.55	1.3	2.08	2.8	3.67	3.79	3.22	2.02	2.56	2.92	4.23	3.27	4.07
Guanine nucleotide-binding protein G(I)/G(S)/G(T) subunit beta-2	1	0	1.67	5.64	6.34	5.95	6.3	6.64	3.13	2.98	2.85	2.4	6.53	4.44
Heat shock protein 105 kDa	1	1.53	0.45	7.05	5.55	2.88	2.96	2.51	3.1	3.29	3.27	2.35	5.63	0.58
Histone deacetylase 2	1	1.3	2.81	6.61	4.47	5.33	1.23	4.93	0.86	4.78	12.24	4.97	7.47	17.55
Inactive tyrosine-protein kinase 7	1	1.03	2.42	6.16	5.42	5.45	5.02	2.12	1.17	1.89	1.47	1.95	1.37	2.31
Kinase non-catalytic C-lobe domain-containing protein 1	1	1.84	3.43	4.16	8.12	10.73	11.48	4.2	2.92	3.99	1.86	6.06	4.34	5.94
Mitochondrial 10-formyltetrahydrofolate dehydrogenase	1	1.62	2.52	7.29	3.88	3.52	2.71	1.25	1.89	4.5	1.86	8.62	5.59	6.7
Myosin light chain 6B	1	0.67	4.57	8.82	9.47	18.1	10.31	10.15	4.16	6.84	11.38	12.03	5.13	4.06
Phosphatidylinositol 4 5-bisphosphate 3-kinase catalytic subunit gamma isoform	1	1.02	8.18	23.29	12.35	20.99	7.28	16.49	7.26	10.67	5.89	8.09	4.52	5.37
Phosphoglycerate kinase 1	1	1.77	2.33	7.89	3.51	5.14	3.37	4.63	1.51	1.76	1.09	1.88	1.63	1.89
Polypyrimidine tract-binding protein 1	1	0	2.33	12.65	5.91	10.62	10.74	4.75	0.84	1.35	2.05	2.49	0.95	4.22
PRA1 family protein 3	1	1.06	2.12	6.38	5.21	5.08	4.43	11.63	2.74	2.32	2.36	3.69	3.67	1.74
Probable cysteine—tRNA ligase mitochondrial	1	0.53	2.51	4.4	8.58	5.45	6.28	3.4	1.19	1.56	12.14	3.94	4.84	2.22
Protein disulfide-isomerase TMX3	1	1.84	3.31	3.2	3.59	4.4	4.97	1.97	2.01	2.87	0.46	4.65	4.35	5.48
Ras GTPase-activating protein-binding protein 1	1	0.51	1.24	3.37	3.36	7.93	3.58	2.48	2.04	2.14	10.88	6.03	9.61	5.76
Signal peptidase complex catalytic subunit SEC11	1	1.51	2.58	4.18	4.97	2.98	1.02	4	1.9	3.79	3.16	1.9	2.25	4.61
Splicing factor 3A subunit 3	1	1.97	2.17	3.71	3.19	3.16	6.27	2.8	2.54	3.71	3.33	5.71	5.72	5.2
Tectonin beta-propeller repeat-containing protein 2	1	1.33	14.08	7.44	10.52	4.96	13.46	3.05	3.24	2.87	4.45	2.35	3.35	4.31
Thy-1 membrane glycoprotein (Fragment)	1	1.44	1.86	3.69	4.4	6.75	10.35	4.83	3.35	5.05	4.08	11.87	7.24	4.6
Tropomyosin alpha-1 chain	1	1.06	1.66	2.26	2.61	5.92	3.99	3.05	2.92	3.7	3.87	6.11	4.75	4.91
V-type proton ATPase subunit	1	0	26.55	42.08	35.48	14.46	19.29	49.37	20.8	34.02	16.25	49.05	20.67	37.65

**Table 2:** Highest number of associated biological process and strongest relevance to neural differentiation and development.

Name	Accession	Gene	Go Biological Process
Adapter molecule crk	P46108	CRK	Cellular response to nerve growth factor stimulus, cerebellar neuron development, cerebral cortex development, dendrite development establishment of cell polarity, hippocampus development, negative regulation of cell motility
Kinase non-catalytic C-lobe domain-containing protein 1	Q76NI1	KNDC1	Cerebellar granule cell differentiation, regulation of dendrite development, regulation of dendrite morphogenesis
Endoplasmic reticulum chaperone BiP	P11021	BIP	Cerebellar Purkinje cell layer development, cerebellum structural organisation, negative regulation of apoptotic process, neuron apoptotic process, neuron differentiation, positive regulation of cell migration, positive regulation of neuron projection development
Inactive tyrosine-protein kinase 7	Q13308	PTK7	Actin cytoskeleton reorganisation, establishment of planar polarity, planar cell polarity pathway involved in neural tube closure, positive regulation of canonical Wnt signalling pathway, positive regulation of neuron projection development
Histone deacetylase 2	Q92769	HDAC2	Cellular response to dopamine, dendrite development, negative regulation of apoptotic process, negative regulation of dendritic spine development, negative regulation of neuron projection development, positive regulation of cell population proliferation
Neurofilament light polypeptide	P07196	NFL	Cerebral cortex development, hippocampus development, intermediate filament organisation, intermediate filament polymerisation or depolymerisation, microtubule cytoskeleton organisation, negative regulation of neuron apoptotic process, neurofilament bundle assembly, neurofilament cytoskeleton organisation, neuron projection morphogenesis, peripheral nervous system axon regeneration, positive regulation of axonogenesis, regulation of axon diameter, spinal cord development, synapse maturation
Neuropilin-2	O60462	NRP2	Axon extension involved in axon guidance, axon guidance, dorsal root ganglion morphogenesis, nerve development, neural crest cell migration involved in autonomic nervous system development, regulation of postsynapse organisation, semaphorin-plexin signalling pathway involved in neuron projection guidance, sensory neuron axon guidance, sympathetic ganglion development, sympathetic neuron projection extension, sympathetic neuron projection guidance
CDK5 regulatory subunit-associated protein 2	Q96SN8	CK5P2	Brain development, microtubule bundle formation, microtubule cytoskeleton organisation, microtubule organising centre organisation, negative regulation of centriole replication, negative regulation of neuron differentiation, neurogenesis, positive regulation of microtubule polymerisation, regulation of neuron differentiation
Hypoxanthine-guanine phosphoribosyltransferase	P00492	HPRT	Central nervous system neuron development, cerebral cortex neuron differentiation, dendrite morphogenesis, dopamine metabolic process, positive regulation of dopamine metabolic process, striatum development
Pituitary adenylate cyclase-activating polypeptide	P18509	PACA	Negative regulation of cell cycle, negative regulation of glial cell proliferation, neuron projection development, neuropeptide signalling pathway, pituitary gland development, positive regulation of cell population proliferation, positive regulation of neuron projection development, regulation of oligodendrocyte progenitor proliferation, regulation of postsynaptic membrane potential
Cadherin-2	P19022	CADH2	Brain morphogenesis, cell migration, cell morphogenesis, cerebral cortex development, glial cell differentiation, negative regulation of canonical Wnt signaling pathway, neuroepithelial cell differentiation, neuroligin clustering involved in postsynaptic membrane assembly, neuronal stem cell population maintenance, positive regulation of synaptic vesicle clustering, radial glial cell differentiation, regulation of axonogenesis, regulation of oligodendrocyte progenitor proliferation, regulation of synaptic transmission, glutamatergic, synapse assembly
GTPase Hras	P01112	RASH	Cell cycle arrest, cell population proliferation, negative regulation of cell population proliferation, negative regulation of gene expression, negative regulation of neuron apoptotic process, positive regulation of actin cytoskeleton reorganisation, positive regulation of cell migration, positive regulation of cell population proliferation, regulation of long-term neuronal synaptic plasticity, regulation of neurotransmitter receptor localisation to postsynaptic specialisation membrane
DNA topoisomerase 2-beta	Q02880	TOP2B	Axonogenesis, forebrain development, neuron migration

**Table 3:** Proteins upregulated higher than two- or fivefold at IBMX-treated cell timepoints and proteins uniquely expressed in treated cells relative to control. For proteins present in controls and treatments expression ratios were calculated. Each column shows treatment concentration and relative timepoint group.

	Controls		6 h				12 h				24 h			
Proteins with Fold Change Relative to Control	FBS	DMEM	0.25 mM	0.5 mM	1 mM	2.5 mM	0.25 mM	0.5 mM	1 mM	2.5 mM	0.25 mM	0.5 mM	1 mM	2.5 mM
Adapter molecule crk	1	1.06	1.61	2.24	3.19	2.56	2.61	0.89	1.14	1.17	1.19	1.25	2.09	2.94
Kinase non-catalytic C-lobe domain-containing protein 1	1	1.84	3.43	4.16	8.12	10.73	11.48	4.2	2.92	3.99	1.86	6.06	4.34	5.94
Endoplasmic reticulum chaperone BiP	1	0.76	1.09	1.95	1.63	2.37	2.02	2.52	1.61	2	2.57	2.83	1.89	1.91
Inactive tyrosine-protein kinase 7	1	1.03	2.42	6.16	5.42	5.45	5.02	2.12	1.17	1.89	1.47	1.95	1.37	2.31
Histone deacetylase 2	1	1.3	2.81	6.61	4.47	5.33	1.23	4.93	0.86	4.78	12.24	4.97	7.47	17.55
No. of proteins above twofold increase	**0**	**0**	**3**	**4**	**4**	**5**	**4**	**4**	**1**	**3**	**2**	**3**	**3**	**4**
No. of proteins above fivefold increase	**0**	**0**	**0**	**2**	**2**	**3**	**2**	**0**	**0**	**0**	**1**	**1**	**1**	**2**
Uniquely expressed in IBMX-treated cells														
Neurofilament light polypeptide	✕	✕	✕	✕	✕	✕	✕	✕	✕	✓	✓	✓	✓	✓
Neuropilin-2	✕	✕	✕	✕	✕	✓	✕	✕	✕	✓	✕	✕	✕	✓
CDK5 regulatory subunit-associated protein 2	✕	✕	✓	✓	✓	✓	✓	✓	✓	✓	✓	✓	✓	✕
Hypoxanthine-guanine phosphoribosyltransferase	✕	✕	✓	✓	✓	✓	✕	✕	✓	✓	✓	✓	✓	✕
Pituitary adenylate cyclase-activating polypeptide	✕	✕	✓	✓	✓	✕	✓	✓	✕	✕	✕	✕	✕	✕
Cadherin-2	✕	✕	✓	✓	✓	✕	✓	✓	✓	✓	✓	✓	✕	✓
GTPase Hras	✕	✕	✓	✓	✓	✓	✕	✓	✓	✓	✓	✓	✓	✕
DNA topoisomerase 2-beta	✕	✕	✓	✓	✓	✓	✕	✓	✓	✓	✓	✓	✓	✓
No. of proteins uniquely expressed in IBMX-treated cells			6	6	6	5	3	5	5	7	6	6	5	4
Total number of significant upregulated proteins post IBMX treatment			**9**	**13**	**12**	**13**	**9**	**9**	**6**	**10**	**9**	**10**	**9**	**10**

The presence of the unique protein is designated by a tick (✓); If the same protein was not detected in other treatments it is designated by a cross (✕).

## Supplementary Materials

Supplementary materials can be found at https://www.mdpi.com/1422-0067/21/14/4867/s1.

## Abbreviations

ADSCs	Adipose-derived stem cells
ALP	Alkaline phosphatase
AVIL	Advillin
bFGF	Basic fibroblast growth factor
BHA	Butylated hydroxyanisole
BIP	Endoplasmic reticulum chaperone BiP
BME	Beta-mercaptoethanol
CADH2	Cadherin-2
cAMP	Cyclic adenosine monophosphate
CK5P2	CDK5 regulatory subunit-associated protein 2
CRK	Adapter molecule crk
D-MEM	Dulbecco’s Modified Eagle Medium: Nutrient Mixture F-12
DMSO	Dimethylsulfoxide
ERK	Extracellular receptor kinase
hADSCs	Human adipose-derived stem cells
HDAC	Histone deacetylase
HDAC2	Histone deacetylase 2
HPRT	Hypoxanthine-guanine phosphoribosyltransferase
IBMX	Isobuytlmethyl xanthine
JAK	Janus kinase
KNDC1	Kinase non-catalytic C-lobe domain-containing protein 1
LC-MS/MS	Liquid chromatography tandem mass spectrometry
MAPK	Mitogen-activated protein kinase
MAPK1	Mitogen-activated protein kinase 1
MSC	Mesenchymal stem cells
NFL	Neurofilament light polypeptide
NRP2	Neuropilin-2
NSE	Neuron-specific enolase
PACA	Pituitary adenylate cyclase-activating polypeptide
PBS	Phosphate-buffered saline
PI3K	Phosphatidylinositol 3-kinase
PTK7	Inactive tyrosine-protein kinase 7
RASH	GTPase Hras
STAT1	Signal transducer and activator of transcription 1-alpha/beta
STAT6	Signal transducer and activator of transcription 6
TOP2B	DNA topoisomerase 2-beta
VA	Valproic acid
VEGF	Vascular endothelial growth factor
