# Supplementary material for: Molecular Mechanisms Involved in Neural Substructure Development during Phosphodiesterase Inhibitor Treatment of Mesenchymal Stem Cells
Source: Int J Mol Sci. 2020 Jul 9;21(14):4867. doi: 10.3390/ijms21144867 (PMC7402296; doi:10.3390/ijms21144867)
Supplement: Supplementary file 1 [file ijms-21-04867-s001.pdf]

Supplementary material

Python Script constructed for data analysis for section 4.7 in methodology.

```
import pandas as pd
from pandas import read_excel
import openpyxl as xl
import xlsxwriter
from xlrd import open_workbook
import pandas
from openpyxl import load_workbook
import re
import numpy as np

global content9
global path1
global column_letter_protein_names
protein_names = "Protein names"
gene_ontology = "Gene ontology (biological process)"
sheet_0 = 'Sheet0'



def make_list_files():
    fname = 'excelfilelist.txt'
    #list of excel files to process, listed as filename.xlsx, each on a new line
    with open(fname) as f:
        global content9
        content9 = f.readlines() #for every new line in text file, make an item in a list
    content9 = [j.strip() for j in content9] #then strip extra spaces for that item

def make_list():
    fname = 'inputwordlist.txt' #list of words you want to search for, each on a new line
    with open(fname) as f:
        global content
        content = f.readlines() #for every new line in text file, make an item in a list
    content = [j.strip() for j in content] #then strip extra spaces for that item


def get_column_letter_protein_names():
    row = sheet.row(0)
    for col_id, cell in enumerate(row): #for every item in the first row
        if cell.value == protein_names :
            #check the column label matches "Protein names"
            global column_letter_protein_names
            column_letter_protein_names = xlsxwriter.utility.xl_col_to_name(col_id)
            #if it does, find the excel column letter
            print ('Printing ' + protein_names + ' from column: ' + column_letter_protein_names)

def get_column_letter_gene_ontology():
    row = sheet.row(0)
```

```
    for col_id, cell in enumerate(row): #for every item in the first row
        if cell.value == gene_ontology : #check the column label matches "Gene ontology (biological process)"
            global column_letter_gene_ontology
            column_letter_gene_ontology = xlsxwriter.utility.xl_col_to_name(col_id)
            #if it does, find the excel column letter
            print ('Printing ' + gene_ontology + ' from column: ' + column_letter_gene_ontology)



def copy_proteins():
    for i in ws1[column_letter_protein_names + ':' + column_letter_protein_names]:
        print('printing from ' + str(i.column) + str(i.row))
        ws2.cell(row=i.row, column=1, value=i.value)
        #basically copy names of proteins to first column

def copy_matched_processes():
    for i in ws1[column_letter_gene_ontology + ':' + column_letter_gene_ontology]:
        print('printing from ' + str(i.column) + str(i.row))
        my_regex = r"([^;]*)(\w*" + re.escape(word_type) + r"\w*)([^[]*)"
        pattern = re.compile(my_regex)
        temp1=str(i.value)
        temp2 = pattern.findall(temp1)
        temp3 = (",".join(map("".join,temp2)))
        temp4 = temp3.strip()
        ws2.cell(row=i.row, column=2, value=temp4)
        print(temp3)
        #copy biological processes that match search term for each protein

def copy_matched_processes_all():
    for i in ws1[column_letter_gene_ontology + ':' + column_letter_gene_ontology]:
        print('printing from ' + str(i.column) + str(i.row))
        my_regex = r"([^;]*)(\w*\w*)([^[]*)"
        pattern = re.compile(my_regex)
        temp1=str(i.value)
        temp2 = pattern.findall(temp1)
        temp3 = (",".join(map("".join,temp2)))
        temp4 = temp3.strip()
        ws2.cell(row=i.row, column=2, value=temp4)
        print(temp3)
        #special case where we copy all biological processes instead of ones that match searched words

def save_first_sheet():
    ws2.cell(row=1, column=1).value = 'protein_names'
    ws2.cell(row=1, column=2).value = 'gene_ontology'
    wb1.save(path1)
    print('Word: \"' + word_type + '\" filtered list of Biological Processes saved.')
    #saves the sheet with protein names and biological processes

def split_by_comma():
    global df1
```

```
    df = read_excel(path1, sheet_name = word_type)
    j = df['gene_ontology'].astype(str)
    s = j.str.split(',').apply(pd.Series, 1).stack()
    s.index = s.index.droplevel(-1)
    s.name = 'gene_ontology'
    del df['gene_ontology']
    df = df.drop(df.index[0], inplace=False)
    df4 = df.join(s)
    df5 = df4.replace(['None,', 'nan','None',np.nan], 'NaN', inplace=False)
    df6 = df5.astype(str)
    df1 = df6[df6.gene_ontology != 'NaN']
    df1 = df1.drop_duplicates()
    print(df1)
    print('Word: \"' + word_type + '\" separated into new rows by comma.')
    #separates biological processes each into their own row, each next to its originating protein
    # then removes duplicates

def split_by_semicolon():
    global df9
    df = read_excel(path1, sheet_name = 'all')
    s = df['gene_ontology'].str.split(';').apply(pd.Series, 1).stack()
    s.index = s.index.droplevel(-1)
    s.name = 'gene_ontology'
    del df['gene_ontology']
    dfa = df.join(s)
    dfb = dfa.replace(['None,', 'nan','None',np.nan], 'NaN', inplace=False)
    dfc = dfb.astype(str)
    df9 = dfc[dfc.gene_ontology != 'NaN']
    df9 = df9.drop_duplicates()
    print(df9)
    print('Word: \"' + word_type + '\" separated into new rows by comma.')
    #special case for the sheet that has all biological processes and not just by search term
    #separates biological processes each into their own row, each next to its originating protein
    # then removes duplicates

def save_second_sheet():
    book = load_workbook(path1)
    writer = pandas.ExcelWriter(path1, engine='openpyxl')
    writer.book = book
    writer.sheets = dict((ws.title, ws) for ws in book.worksheets)
    df1.to_excel(excel_writer = writer, sheet_name = word_type + "_a", index=False, columns=['protein_names', 'gene_ontology'])
    writer.save()
    print('Word: \"' + word_type + '\" table saved.')
    #saves these sheets

def save_second_sheet_all():
    book = load_workbook(path1)
    writer = pandas.ExcelWriter(path1, engine='openpyxl')
    writer.book = book
    writer.sheets = dict((ws.title, ws) for ws in book.worksheets)
    df9.to_excel(excel_writer = writer,sheet_name = "all_a", index=False, columns=['protein_names', 'gene_ontology'])
```

```
    writer.save()
    print('Word: \"' + word_type + '\" table saved.')
    #speciual case to save sheet for all biological processes

def count_proteins():
    global df2
    k = pd.read_excel(path1,sheet_name = word_type +'_a')['protein_names']
    df2 = k.value_counts()
    print(df2)
    book = load_workbook(path1)
    writer = pandas.ExcelWriter(path1, engine='openpyxl')
    writer.book = book
    writer.sheets = dict((ws.title, ws) for ws in book.worksheets)
    df2.to_excel(writer,word_type + "_b")
    writer.save()
    #counts how many rows we have in the second sheet,
    #enumerating how many proteins we have per search term

def count_processes():
    global df3
    j = pd.read_excel(path1,sheet_name = word_type +'_a')['gene_ontology']
    df3 = j.value_counts()
    print(df3)
    book = load_workbook(path1)
    writer = pandas.ExcelWriter(path1, engine='openpyxl')
    writer.book = book
    writer.sheets = dict((ws.title, ws) for ws in book.worksheets)
    df3.to_excel(writer,word_type + "_c")
    writer.save()
    #counts how many rows we have in the second sheet,
    #enumerating how many biological processes we have per search term

#order we run the functions
make_list_files()
for i in (range(len(content9))):
    path1 = (content9[i])
    wb1 = xl.load_workbook(filename=path1)
    ws1 = wb1.worksheets[0]
    book = open_workbook(path1)
    sheet = book.sheet_by_name(sheet_0)
    make_list()
    word_type = ('all')
    ws2 = wb1.create_sheet(word_type)
    get_column_letter_protein_names()
    copy_proteins()
    get_column_letter_gene_ontology()
    copy_matched_processes_all()
    save_first_sheet()
    for y in (range(len(content))):
        word_type = (content[y])
        ws2 = wb1.create_sheet(word_type)
        get_column_letter_protein_names()
        copy_proteins()
        get_column_letter_gene_ontology()
        copy_matched_processes()
        save_first_sheet()
    for z in (range(len(content))):
        word_type = (content[z])
```

```
        split_by_comma()
        save_second_sheet()
    split_by_semicolon()
    save_second_sheet_all()
    content.insert(0,'all')
    for v in (range(len(content))):
        word_type = (content[v])
        count_proteins()
    for u in (range(len(content))):
        word_type = (content[u])
        count_processes()
```

Supplementary Table 1 The gene ontology of proteins identified in biological processes with neural associations across dataset.

GO Term	GO:ID	Percentage Associated Proteins Found	Number of Proteins	Term P-Value
neurogenesis	GO:0022008	10.41	214	8.18E-06
generation of neurons	GO:0048699	10.46	202	1.07E-05
positive regulation of cell differentiation	GO:0045597	11.88	152	1.39E-07
neuron differentiation	GO:0030182	10.54	135	2.91E-04
neuron development	GO:0048666	11.40	121	2.06E-05
regulation of neurogenesis	GO:0050767	10.79	116	3.40E-04
neuron projection development	GO:0031175	12.43	109	1.13E-06
regulation of neuron differentiation	GO:0045664	11.14	97	3.59E-04
regulation of neuron projection development	GO:0010975	12.63	84	1.22E-05
positive regulation of neurogenesis	GO:0050769	12.62	82	1.42E-05
neuron projection morphogenesis	GO:0048812	12.52	81	2.59E-05
positive regulation of MAPK cascade	GO:0043410	9.66	71	0.067723119
axon development	GO:0061564	13.16	70	1.70E-05
positive regulation of neuron differentiation	GO:0045666	12.84	67	6.22E-05
cell morphogenesis involved in neuron differentiation	GO:0048667	11.78	67	8.05E-04
MAPK cascade	GO:0000165	13.09	64	5.11E-05
regulation of neuron death	GO:1901214	14.96	63	5.15E-07
axonogenesis	GO:0007409	12.78	62	1.43E-04
positive regulation of neuron projection development	GO:0010976	15.02	61	7.86E-07
regulation of synapse structure or activity	GO:0050803	15.99	51	8.56E-07
regulation of synapse organization	GO:0050807	16.61	51	2.61E-07
regulation of neuron apoptotic process	GO:0043523	17.69	49	5.59E-08
regulation of neurotransmitter levels	GO:0001505	10.31	46	0.049550793
axon guidance	GO:0007411	13.64	45	2.52E-04
neuron projection guidance	GO:0097485	13.47	45	4.07E-04
negative regulation of neuron death	GO:1901215	15.60	44	9.48E-06
negative regulation of neurogenesis	GO:0050768	10.96	41	0.031487044

positive regulation of MAP kinase activity	GO:0043406	10.43	39	0.063876255
negative regulation of neuron apoptotic process	GO:0043524	18.00	36	2.05E-06
regulation of axonogenesis	GO:0050770	14.63	36	2.71E-04
positive regulation of ERK1 and ERK2 cascade	GO:0070374	11.54	33	0.025505733
negative regulation of neuron differentiation	GO:0045665	10.85	32	0.061859457
regulation of synaptic plasticity	GO:0048167	11.65	29	0.031369787
glial cell differentiation	GO:0010001	10.64	25	0.110965306
negative regulation of neuron projection development	GO:0010977	12.76	25	0.015134848
regulation of axon extension	GO:0030516	20.66	25	5.96E-06
positive regulation of axonogenesis	GO:0050772	20.83	25	5.10E-06
neuron migration	GO:0001764	14.65	23	0.003883542
central nervous system neuron differentiation	GO:0021953	8.33	21	0.722944115
neuron projection extension	GO:1990138	23.60	21	3.66E-06
positive regulation of neuron death	GO:1901216	16.26	20	0.001844229
positive regulation of axon extension	GO:0045773	28.36	19	5.27E-07
neurotransmitter transport	GO:0006836	8.00	18	0.900360041
glial cell development	GO:0021782	11.46	18	0.098474084
response to axon injury	GO:0048678	25.00	17	1.34E-05
neuromuscular process	GO:0050905	10.69	17	0.1800346
axon extension	GO:0048675	30.19	16	1.65E-06
neuron recognition	GO:0008038	20.00	14	9.07E-04
positive regulation of neuron apoptotic process	GO:0043525	19.72	14	0.001049444
astrocyte differentiation	GO:0048708	17.28	14	0.005175463
regulation of synapse assembly	GO:0051963	10.77	14	0.191534506
axonal transport	GO:0098930	16.87	14	0.005944255
neuromuscular junction development	GO:0007528	18.57	13	0.002743423
central nervous system neuron development	GO:0021954	11.50	13	0.156246699
neuromuscular process controlling balance	GO:0050885	14.61	13	0.02665154
phosphatidylinositol metabolic process	GO:0046488	5.97	12	0.426269868
regulation of neurotransmitter transport	GO:0051588	6.94	12	0.776348742

astrocyte development	GO:0014002	18.64	11	0.005479412
neurotransmitter metabolic process	GO:0042133	9.48	11	0.485807053
negative regulation of axonogenesis	GO:0050771	11.70	11	0.173288857
regulation of neurotransmitter receptor activity	GO:0099601	9.73	11	0.478815666
neuron projection organization	GO:0106027	16.92	11	0.016409561
neurotransmitter secretion	GO:0007269	8.00	10	0.867413349
ensheathment of neurons	GO:0007272	6.13	10	0.55614281
anterograde axonal transport	GO:0008089	15.15	10	0.036115029
glial cell migration	GO:0008347	16.67	10	0.024648435
axon ensheathment	GO:0008366	6.13	10	0.55614281
forebrain generation of neurons	GO:0021872	12.66	10	0.135270336
regulation of synaptic transmission, glutamatergic	GO:0051966	11.49	10	0.224224463
neuron death	GO:0070997	14.29	10	0.067924084
regulation of postsynaptic membrane neurotransmitter receptor levels	GO:0099072	14.49	10	0.065939583
phosphatidylinositol biosynthetic process	GO:0006661	6.92	9	0.86951678
motor neuron axon guidance	GO:0008045	19.57	9	0.008365169
neuron projection regeneration	GO:0031102	25.71	9	0.001164549
axon regeneration	GO:0031103	33.33	9	1.37E-04
neurotrophin signaling pathway	GO:0038179	19.15	9	0.009646455
neuron apoptotic process	GO:0051402	15.25	9	0.046781029
neuroblast proliferation	GO:0007405	19.51	8	0.012811679
regulation of neurotransmitter secretion	GO:0046928	6.11	8	0.622536021
regulation of neuron migration	GO:2001222	12.70	8	0.154017294
neuropeptide signaling pathway	GO:0007218	5.30	7	0.331197706
retrograde axonal transport	GO:0008090	23.33	7	0.007242808
forebrain neuron differentiation	GO:0021879	11.86	7	0.224660544
forebrain neuron development	GO:0021884	21.88	7	0.010445554
telencephalon glial cell migration	GO:0022030	17.07	7	0.037940934
regulation of neuronal synaptic plasticity	GO:0048168	10.14	7	0.495189522
glial cell activation	GO:0061900	12.50	7	0.205025062

neurotransmitter receptor transport	GO:0099637	14.89	7	0.092783965
neuroinflammatory response	GO:0150076	11.86	7	0.224660544
semaphorin-plexin signaling pathway involved in neuron projection guidance	GO:1902285	35.00	7	5.50E-04
neuron projection maintenance	GO:1990535	29.17	7	0.00185991
axonal fasciculation	GO:0007413	17.14	6	0.051917636
negative regulation of axon extension	GO:0030517	12.50	6	0.271232909
regulation of glial cell differentiation	GO:0045685	6.45	6	0.845542224
neurotrophin TRK receptor signaling pathway	GO:0048011	19.35	6	0.030644531
neuroepithelial cell differentiation	GO:0060563	9.68	6	0.483198865
neuron cellular homeostasis	GO:0070050	12.00	6	0.282756046
neuron projection fasciculation	GO:0106030	17.14	6	0.051917636
semaphorin-plexin signaling pathway involved in axon guidance	GO:1902287	33.33	6	0.001845789
regulation of oxidative stress-induced neuron death	GO:1903203	15.38	6	0.122041196
positive regulation of neuron migration	GO:2001224	21.43	6	0.019132265
neuropeptide receptor activity	GO:0008188	8.77	5	0.802389973
cerebral cortex neuron differentiation	GO:0021895	12.50	5	0.238332257
retinal ganglion cell axon guidance	GO:0031290	19.23	5	0.048070584
neurotransmitter biosynthetic process	GO:0042136	8.33	5	0.808932146
astrocyte activation	GO:0048143	16.13	5	0.090373018
regulation of long-term neuronal synaptic plasticity	GO:0048169	13.16	5	0.217679369
axon extension involved in axon guidance	GO:0048846	41.67	5	0.001440813
regulation of synapse maturation	GO:0090128	18.52	5	0.055343951
motor neuron migration	GO:0097475	41.67	5	0.001440813
neurotransmitter receptor transport to plasma membrane	GO:0098877	18.52	5	0.055343951
neurotransmitter receptor transport, endosome to postsynaptic membrane	GO:0098887	27.78	5	0.010503591
neurotransmitter receptor transport to postsynaptic membrane	GO:0098969	18.52	5	0.055343951
neurotransmitter receptor transport, endosome to plasma membrane	GO:0099639	27.78	5	0.010503591
neuron projection extension involved in neuron projection guidance	GO:1902284	41.67	5	0.001440813
negative regulation of oxidative stress-induced neuron death	GO:1903204	16.67	5	0.080739391
neurotransmitter uptake	GO:0001504	8.89	4	0.777762825

microglial cell activation	GO:0001774	9.52	4	0.567656276
astrocyte activation involved in immune response	GO:0002265	33.33	4	0.011062181
neuropeptide hormone activity	GO:0005184	10.00	4	0.551274934
peripheral nervous system axon regeneration	GO:0014012	57.14	4	0.001074355
cerebral cortex tangential migration using cell-axon interactions	GO:0021824	57.14	4	0.001074355
gonadotrophin-releasing hormone neuronal migration to the hypothalamus	GO:0021828	57.14	4	0.001074355
hypothalamic tangential migration using cell-axon interactions	GO:0021856	57.14	4	0.001074355
hypothalamus gonadotrophin-releasing hormone neuron differentiation	GO:0021886	50.00	4	0.002015776
hypothalamus gonadotrophin-releasing hormone neuron development	GO:0021888	50.00	4	0.002015776
central nervous system neuron axonogenesis	GO:0021955	7.55	4	1
neuronal stem cell division	GO:0036445	25.00	4	0.03166553
neuron maturation	GO:0042551	7.02	4	1
regulation of axon extension involved in axon guidance	GO:0048841	11.43	4	0.349042389
negative regulation of axon extension involved in axon guidance	GO:0048843	13.79	4	0.281813477
regulation of neurotransmitter uptake	GO:0051580	16.00	4	0.126897094
neuroblast division	GO:0055057	25.00	4	0.03166553
axonogenesis involved in innervation	GO:0060385	36.36	4	0.007855058
regulation of neuron projection regeneration	GO:0070570	9.52	4	0.567656276
commissural neuron axon guidance	GO:0071679	22.22	4	0.047034962
neuroligin clustering involved in postsynaptic membrane assembly	GO:0097118	36.36	4	0.007855058
sensory neuron axon guidance	GO:0097374	57.14	4	0.001074355
sympathetic neuron projection extension	GO:0097490	50.00	4	0.002015776
sympathetic neuron projection guidance	GO:0097491	50.00	4	0.002015776
regulation of neuroinflammatory response	GO:0150077	8.33	4	0.787838259
regulation of axon guidance	GO:1902667	8.16	4	0.791680328
negative regulation of axon guidance	GO:1902668	12.50	4	0.311872855
phosphatidylinositol 3-kinase signaling	GO:0014065	6.98	3	1
phosphatidylinositol phosphate kinase activity	GO:0016307	17.65	3	0.142872959
axonal transport of mitochondrion	GO:0019896	12.00	3	0.440814094
spinal cord association neuron differentiation	GO:0021527	15.00	3	0.202403131

branchiomotor neuron axon guidance	GO:0021785	30.00	3	0.037733139
regulation of synaptic transmission, GABAergic	GO:0032228	6.82	3	1
peripheral nervous system axon ensheathment	GO:0032292	8.33	3	0.758070158
neuropilin signaling pathway	GO:0038189	37.50	3	0.019808704
VEGF-activated neuropilin signaling pathway	GO:0038190	60.00	3	0.00422891
neurotransmitter catabolic process	GO:0042135	13.64	3	0.244763095
positive regulation of glial cell differentiation	GO:0045687	5.17	3	0.62507609
phosphatidylinositol phosphorylation	GO:0046854	5.26	3	0.62468046
axon extension involved in regeneration	GO:0048677	100.00	3	4.77E-04
regulation of axon regeneration	GO:0048679	8.11	3	0.764252827
sprouting of injured axon	GO:0048682	100.00	3	4.77E-04
positive regulation of axon extension involved in axon guidance	GO:0048842	27.27	3	0.04893742
positive regulation of neurotransmitter transport	GO:0051590	5.66	3	0.797111188
Bergmann glial cell differentiation	GO:0060020	30.00	3	0.037733139
regulation of glial cell proliferation	GO:0060251	7.89	3	1
dopaminergic neuron differentiation	GO:0071542	7.50	3	1
regulation of retinal ganglion cell axon guidance	GO:0090259	33.33	3	0.028010774
neurotransmitter receptor transport postsynaptic membrane to endosome	GO:0098968	37.50	3	0.019808704
protein localization to axon	GO:0099612	25.00	3	0.0615625
anterograde axonal protein transport	GO:0099641	23.08	3	0.075529828
neuron projection arborization	GO:0140058	16.67	3	0.162040558
positive regulation of retinal ganglion cell axon guidance	GO:1902336	60.00	3	0.00422891
VEGF-activated neuropilin signaling pathway involved in axon guidance	GO:1902378	75.00	3	0.001796256
positive regulation of axon guidance	GO:1902669	25.00	3	0.0615625
regulation of oxidative stress-induced neuron intrinsic apoptotic signaling pathway	GO:1903376	33.33	3	0.028010774
